# Case Report: Synchronous primary lung and thyroid cancers – management informed by multimodal imaging and pathologic correlation

**DOI:** 10.3389/fonc.2026.1776984

**Published:** 2026-04-23

**Authors:** Meng Yuan, Weiwei Rui, Fan Fu, Mengyi Pan, Yun Xi

**Affiliations:** 1 Department of Nuclear Medicine, Ruijin Hospital, Shanghai Jiao Tong University School of Medicine, Shanghai, China; 2 Department of Pathology, Ruijin Hospital, Shanghai Jiao Tong University School of Medicine, Shanghai, China

**Keywords:** ^131^I SPECT, ^18^F-FDG PET/CT, differentiated thyroid carcinoma, lung cancer, therapeutic management

## Abstract

Management of recurrent thyroid cancer with concurrent pulmonary lesions remains challenging because of tumor heterogeneity. Here, we demonstrate that dual-modality imaging—comprising ^18^F-fluorodeoxyglucose (FDG) PET/CT and ^131^I-NaI SPECT/CT is essential for characterizing the metabolic and functional landscape of metastatic disease. Our analysis revealed a significant phenotypic discordance, which includes consistent GLUT1/HK2-driven hypermetabolism contrasted with heterogeneous NIS expression across metastatic lesions. This variability in NIS levels was the primary determinant of divergent RAI uptake and subsequent therapeutic efficacy. These results validate a multimodal approach involving aggressive cytoreductive surgery and high-dose RAI and underscore the importance of identifying molecular drivers of resistance to optimize outcomes in patients with advanced RAI-refractory thyroid cancer.

## Introduction

Differentiated thyroid cancer (DTC) typically follows an indolent clinical course with a favorable prognosis. However, approximately 10–15% of patients develop distant metastases, with pulmonary involvement being the most prevalent, accounting for 50–70% of all metastatic cases (1–3). Notably, the incidence of second primary malignancies (SPMs) in thyroid cancer survivors is significantly higher than that in the general population; the most frequent secondary malignancies include lung, breast, and colorectal cancers (4, 5). This clinical scenario often presents a diagnostic conundrum because of overlapping radiological features and subtle symptomatic presentations. Consequently, early differentiation through advanced imaging modalities—such as PET/CT—and molecular pathological profiling (6, 7) has become critical. The development of individualized, precision-based treatment regimens remains a significant challenge in the contemporary management of complex thyroid cancer presentations.

The diagnostic stratification and therapeutic management of recurrent or metastatic differentiated thyroid cancer (DTC) increasingly rely on multimodal imaging integration and collaborative molecular pathological evaluation (10–12). This approach leverages ^18^F-FDG PET/CT to identify the Warburg effect—mediated by GLUT1 and HK2 overexpression (8, 9)—along with ^131^I-NaI imaging to assess functional iodine uptake facilitated by the sodium–iodide symporter (NIS). In this case, comprehensive Warburg/NIS expression profiling in a patient with postsurgical recurrent thyroid cancer successfully differentiated metastatic lesions from concurrent primary lung adenocarcinoma. Confirming the thyroid origin via total thyroidectomy enabled subsequent high-dose radioactive iodine (RAI) therapy. Furthermore, by prioritizing the management of lung adenocarcinoma on the basis of its superior biological aggressiveness, we optimized the patient’s clinical trajectory. This case highlights a pivotal paradigm for personalized diagnostic workflows and precision oncology in complex metastatic presentations.

## Case presentation

**Figure 1:**
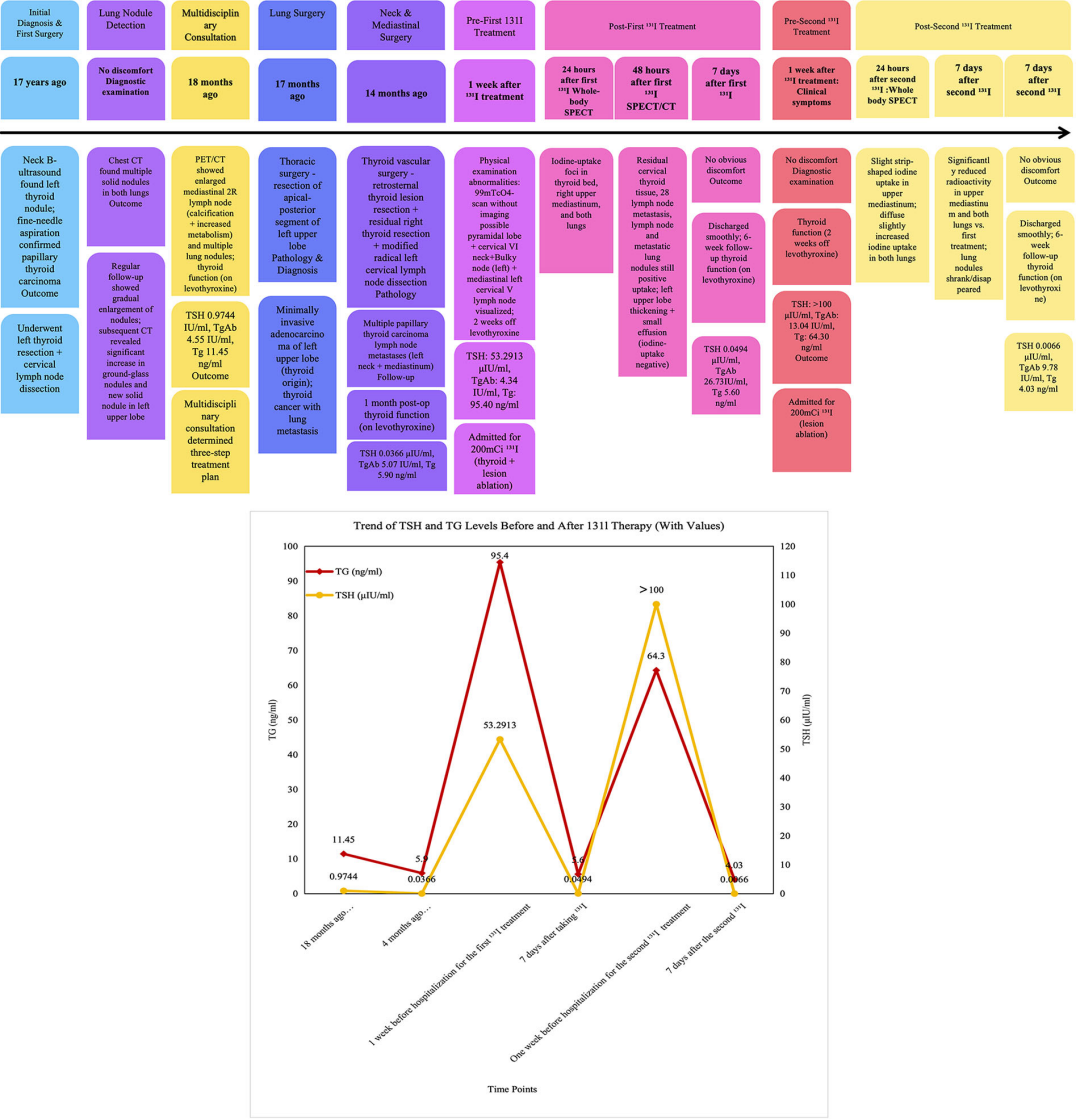
Timeline of the disease course.

The patient, a 46-year-old female, initially underwent radical resection for left-sided thyroid carcinoma in 2007. During the surveillance period five years post-surgery, multiple pulmonary nodules were identified. Subsequent longitudinal follow-up revealed significant enlargement and progression of these lesions, as detailed in the clinical management timeline (**Figure 1**). HRCT on May 31, 2024, revealed bilateral pulmonary nodules, notably a 19×17 mm ground-glass nodule (GGN) in the left upper lobe with peripheral solid components; the other nodules appeared solid. While hilar lymphadenopathy was absent, multiple enlarged mediastinal lymph nodes were detected, including a stable, calcified 2R lesion. Integrated ^18^F-FDG PET/CT suggested synchronous dual primary malignancies. Biochemical markers were a Tg concentration of 11.45 ng/mL and a TGAb concentration of 4.55 IU/mL. The residual right thyroid demonstrated heterogeneous density without hypermetabolism. The patient’s cervical lymphadenopathy was metabolically quiescent. Notably, the 2R mediastinal nodes were hypermetabolic (SUVmax 3.7) and calcified, indicating metastatic DTC. Conversely, a metabolic dichotomy was observed in the lungs: while the solid nodules were non-FDG-avid, the left lingular GGN (2.0×1.4 cm) showed significant uptake (SUVmax 3.6) and was highly suspicious of primary lung adenocarcinoma.

Following the MDT consultation, the patient underwent left upper lobe apical posterior segmentectomy on July 5, 2024. Although initial pathology suggested minimally invasive adenocarcinoma, IHC profiling (Napsin A-/PAX-8+) and clinical history corroborated metastatic thyroid carcinoma. On October 9, 2024, combined thyroid and thoracic surgery confirmed extensive papillary thyroid cancer (PTC) metastases, including anterior superior mediastinal (5/10), middle superior mediastinal (7/7), and left cervical level VI lymph nodes (4/4), with extranodal extension into the perimediastinal fibroadipose tissue. This definitively established systemic multistation metastasis involving the mediastinum, neck, and lungs. Postoperative biochemical surveillance revealed a stimulated Tg concentration of 5.90 ng/mL and a persistently elevated TGAb concentration of 5.07 IU/mL.

**Figure 2:**
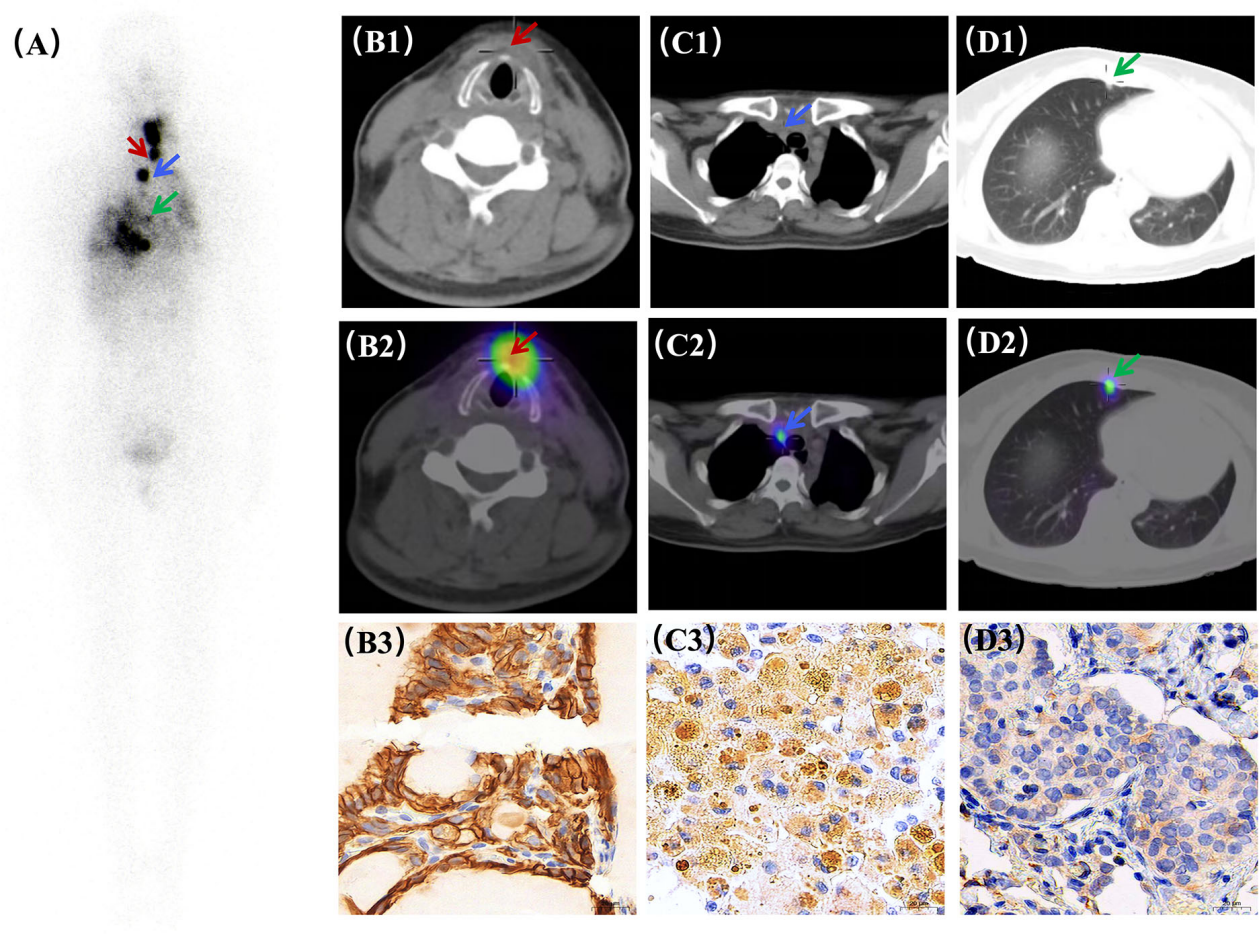
Postoperative ^131^I scintigraphy of thyroid cancer metastasis and validation of NIS protein expression. In the post-therapeutic ^131^I whole-body scan **A**), multiple foci of abnormal radioactive accumulation were identified, representing thyroid cancer metastases, including the residual thyroid bed in the neck, enlarged lymph nodes in the mediastinal 2R station, and bilateral lung parenchyma (consistent with multiple pulmonary nodules). Conversely, physiologic iodine distribution is observed in the nasopharynx, salivary glands, stomach, bladder, and portions of the intestinal tract. Notably, postoperative changes in the left chest (pleural thickening and minor effusion) revealed no ^131^I uptake, further confirming the nonthyroidal nature of these localized reactive changes. **(A)** Whole-body MIP imaging identifying residual thyroid tissue, mediastinal masses, and pulmonary nodules (arrows). **(B1–B3)** Residual thyroid lesions: CT, SPECT, and NIS immunohistochemical staining. **(C1–C3)** Mediastinal lesions: CT, SPECT, and NIS staining. **(D1–D3)** Pulmonary nodules: CT, SPECT, and NIS staining. (**B3–D3**: Magnification, ×400; Scale bar = 20 μm).

Approximately 60 days (two months) after the combined thyroid and thoracic surgery, the patient was admitted to the Nuclear Medicine Department for therapeutic ^131^I administration. Pretreatment biochemical assessment revealed a stimulated Tg of 95.40 ng/mL and a TGAb of 4.34 U/mL. The post-therapeutic ^131^I whole-body scan (WBS) and SPECT/CT fusion imaging (**Figure 2A**) demonstrated no significant thyroid bed uptake. However, multiple RAI-avid metastases were identified, including nodules in the left anterior cervical muscle space (**Figures 2B1, B2**), the right paratracheal VII region, the right subclavian region, and the mediastinal 2R lymph node (**Figures 2C1, C2**), alongside bilateral pulmonary nodules (**Figures 2D1, D2**). Minor left-sided pleural thickening and effusion were noted post-thoracic surgery. At the 60-day follow-up, the suppressed Tg concentration decreased to 5.60 ng/mL, whereas the TGAb concentration increased to 26.73 IU/mL. Comparative IHC analysis of the pulmonary lesions and thyroid metastases revealed the molecular basis for the observed imaging dichotomy (**Figures 2B3, D3**). The diminished ^18^F-FDG uptake in specific thyroid metastases was primarily attributed to GLUT1 silencing and HK2 downregulation, impairing glucose transport. In contrast, primary lung cancer exhibited intense FDG avidity driven by synergistic GLUT1/HK2 overexpression. Furthermore, NIS expression in metastatic foci displayed significant spatial heterogeneity: regions with high NIS levels facilitated ^131^I uptake, whereas low-expression zones indicated potential treatment resistance. The complete absence of NIS was demonstrated in primary lung cancer. This molecular divergence provides a definitive framework for differential diagnosis and personalized theranostics, including the potential for differentiation-inducing agents combined with RAI therapy.

## Discussion

Characteristic CT features of pulmonary metastases from thyroid carcinoma typically include diffuse, miliary-like micronodules (predominantly <1 cm in diameter) with well-defined margins and homogeneous density, often following a pattern of hematogenous or lymphatic dissemination (13). In contrast, primary lung cancer frequently presents as solitary nodules or masses manifesting as ground-glass opacities (GGOs), mixed-density lesions, or solid lesions. These are typically characterized by irregular or poorly defined contours, often accompanied by lobulation, spiculation, or pleural indentation signs (14).

**Figure 3:**
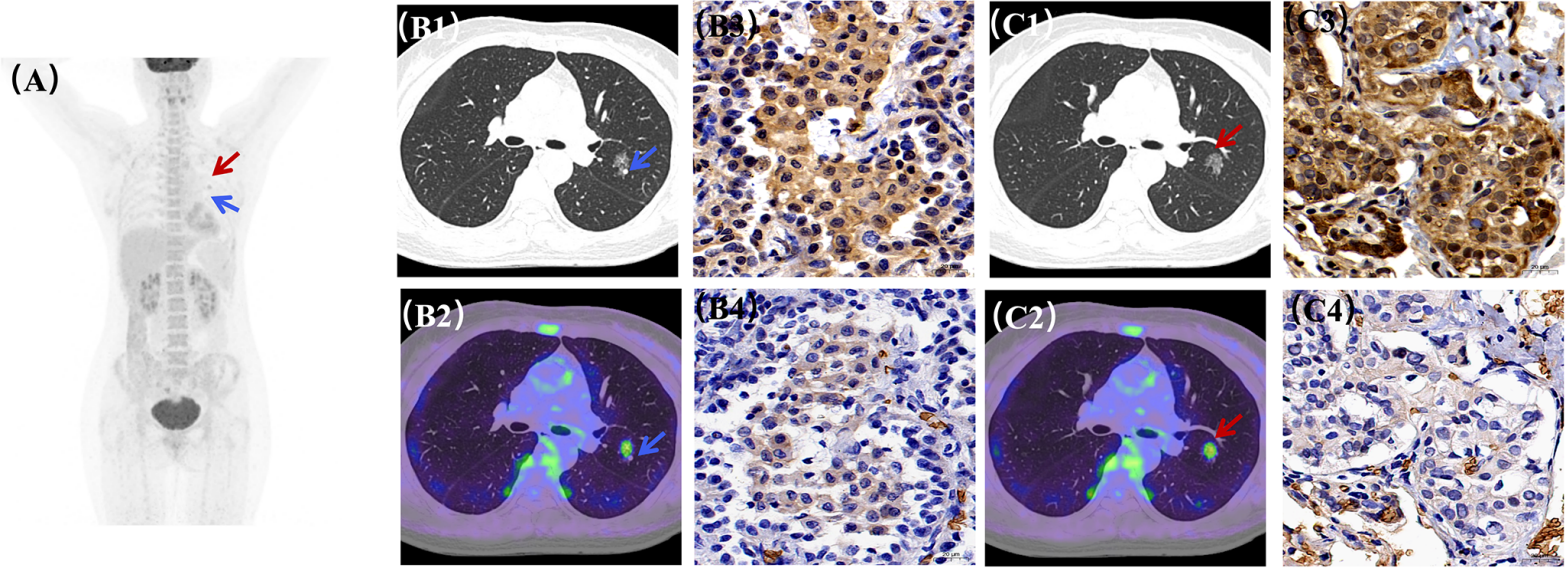
^18^F-FDG metabolic imaging and HK2 and GLUT1 expression in primary and metastatic cancer lesions. PET/CT imaging figure revealed heterogeneous density in the right residual thyroid bed with no significant abnormal FDG uptake (background levels). The enlarged, calcified lymph nodes at the mediastinal 2R station exhibited increased metabolic activity, with an SUVmax of 3.7. Among the multiple pulmonary nodules, solid lesions showed no significant FDG avidity; however, a specific nodule in the left upper lingual segment demonstrated increased metabolism, with an SUVmax of 3.6. **(A)** Whole-body MIP imaging identifying primary lung cancer and metastatic thyroid lesions. **(B1–B4)** Thyroid cancer lung metastasis: CT, fusion PET/CT, HK2 staining, and GLUT1 expression. **(C1–C4)** Primary lung cancer: CT, fusion PET/CT, HK2 staining, and GLUT1 expression. **(B3, B4, C3, C4**: Magnification, ×400; Scale bar = 20 μm).

While dispersed solid pulmonary nodules on CT were characteristic of metastatic thyroid carcinoma, the ground-glass nodule (GGN) with short spicules in the right upper lobe exhibited a distinct radiological phenotype. Multimodal integration of ^18^F-FDG PET/CT, ^131^I imaging, and histopathology proved essential for differential diagnosis. Most primary lung adenocarcinomas demonstrate intense FDG avidity (SUVmax > 5) without ^131^I uptake (**Figures 3A, B1, B2**), influencing subsequent therapeutic stratification (15–17). Conversely, differentiated thyroid cancer (DTC) metastases typically exhibit low FDG affinity but may retain ^131^I avidity (**Figures 3A, C1, C2**) (3, 18–20). Increased FDG uptake in DTCs often signifies dedifferentiation, which can impair iodine transport and diminish the efficacy of RAI therapy. In this case, the hypermetabolic GGN in the right upper lobe, in contrast to the metabolically quiescent solid nodules, suggested a higher malignant potential and a likely synchronous second primary malignancy (SPM). The absence of other hypermetabolic foci on PET/CT indicated early-stage lung cancer, which was later confirmed as adenocarcinoma via surgical pathology. The molecular underpinnings of these metabolic variations involve the differential expression of GLUT1 and HK2; lung cancer tissues exhibited synergistic high expression (+++) of both markers, whereas DTC metastases showed only weak GLUT1 (+) and diminished HK2 levels (**Figures 3B3, B4, C3, C4**).

Furthermore, subsequent ^131^I whole-body imaging confirmed iodine uptake in the remaining nodules, identifying them as DTC metastases. This functional divergence is driven by the sodium–iodide symporter (NIS). NIS protein expression in thyroid cancer metastases demonstrates significant spatial heterogeneity: the NIS-positive rate in metastatic lymph nodes is approximately 60–70% (21), whereas in pulmonary metastases, it typically ranges from 20% to 40% (22). Regions with preserved membrane localization facilitate effective ^131^I influx, whereas those with expression loss or cytoplasmic mislocalization lead to diminished iodine uptake (23). In contrast, primary lung adenocarcinoma typically exhibits negligible NIS expression because of epigenetic silencing, such as promoter methylation (24), resulting in a total lack of ^131^I uptake. However, it is essential to acknowledge that ^131^I avidity is not solely determined by NIS expression; it is also modulated by other critical factors, including the adequacy of TSH stimulation, the degree of lesion vascularity, and competitive inhibition within the systemic iodine pool (25). These physiological determinants, in conjunction with NIS functional integrity, collectively dictate the therapeutic efficacy of radioiodine (RAI) therapy.

Given the biological aggressiveness and limited therapeutic window for early-stage lung cancer, the NCCN guidelines recommend prioritizing the surgical resection of primary lung malignancies when they coexist with thyroid metastases to maximize curative potential (26). Conversely, differentiated thyroid cancer (DTC) is often characterized by an indolent clinical course and protracted progression, allowing it to be managed as a secondary therapeutic target. While ATA guidelines support active surveillance or elective surgery for low-risk DTC (20), this patient presented with high-risk disease, as evidenced by extensive lymphadenopathy and pulmonary involvement. To balance these competing clinical priorities, we implemented a staged multidisciplinary strategy: prioritizing lung cancer resection followed by ^131^I therapy within one year. This sequencing approach was designed to address the most immediate oncological threat while ensuring timely radioactive iodine (RAI) intervention for high-risk DTC, thereby optimizing the patient’s overall prognostic outcome.

NIS expression in metastatic DTCs is significantly heterogeneous and is modulated by the tumor microenvironment and disease progression. Mediastinal lymph node metastases, which benefit from a robust blood supply, maintain higher NIS expression (the literature reports 40%–60% positivity) and substantial iodine avidity (mean SUVmax of 6.2). Conversely, hypoxia-driven downregulation in pulmonary metastases results in decreased NIS expression (20%–40% positivity) and a decreased SUVmax (2.0–3.0) (27). This spatial difference underscores the progressive dedifferentiation during disease progression. Notably, compared with delayed treatment, ^131^I therapy within six months post-thyroidectomy yields a 40% higher NIS positivity rate. The “crossfire effect” from ^131^I cells can eradicate adjacent NIS-negative populations, resulting in a 70%–80% 5-year remission rate for lesions <1 cm. However, for lesions ≥1 cm, remission is less than 20% because of central hypoxia and the limited radiation penetration (28). Consequently, the 2023 EANM guidelines recommend prioritizing surgical resection for large lesions, followed by RAI therapy to eliminate occult micrometastases (29).

Furthermore, postoperative management must address the competing iodine kinetics between residual thyroid tissue and metastatic lesions. Since normal thyroid tissue expresses higher NIS levels, it preferentially sequesters ^131^I, potentially shielding metastases from therapeutic radiation. Excessive thyroid remnants (>5 g) can result in subtherapeutic dosages to metastatic sites (<50 Gy) and increase the risk (1%–3%) of iatrogenic complications, such as severe neck edema or airway compromise, following high-dose ^131^I. Consequently, clinical guidelines emphasize that total thyroidectomy is fundamental to ensuring both therapeutic efficacy and patient safety. In this case, to optimize subsequent ^131^I therapy, the residual right thyroid lobe was resected concurrently with the excision of bulky mediastinal lymph nodes, thereby eliminating iodine competition and mitigating the risk of local radiation-induced complications.

## Conclusions

This case highlights the clinical significance of an integrated diagnostic framework—combining ^18^F-FDG PET/CT with immunohistochemical profiling—to differentiate pulmonary metastases of thyroid origin from primary lung malignancies. Such differentiation is pivotal for therapeutic stratification and for optimizing clinical outcomes. For patients with synchronous dual primary tumors, management strategies must be tailored on the basis of multimodal imaging findings, pathological characteristics, and the projected efficacy of ^131^I therapy. Our findings emphasize that early ^131^I intervention, ideally within one year post-resection, is essential for preserving sodium–iodide symporter (NIS) functionality and maximizing therapeutic potential in patients with metastatic thyroid cancer. This individualized approach provides a robust model for navigating complex oncological scenarios and ensuring timely, evidence-based interventions.

## Data Availability

The original contributions presented in the study are included in the article/**Supplementary Material**. Further inquiries can be directed to the corresponding author.
